# Identifying high quality medical education websites in Otolaryngology: a guide for medical students and residents

**DOI:** 10.1186/s40463-017-0220-4

**Published:** 2017-05-25

**Authors:** Nathan Yang, Sarah Hosseini, Marco A. Mascarella, Meredith Young, Nancy Posel, Kevin Fung, Lily H. P. Nguyen

**Affiliations:** 1 Faculty of Medicine, McGill University, Montreal, QC Canada; 2 Department of Otolaryngology – Head and Neck Surgery, McGill University, Montreal, QC Canada; 3 Center for Medical Education, McGill University, Montreal, QC Canada; 4 Department of Medicine, McGill University, Montreal, QC Canada; 5 McGill Molson Medical Informatics, McGill University, Montreal, QC Canada; 6 Department of Otolaryngology – Head and Neck Surgery, Western University, London, ON Canada

**Keywords:** Medical education, Assessment tool, Online resources

## Abstract

**Background:**

Learners often utilize online resources to supplement formalized curricula, and to appropriately support learning, these resources should be of high quality.﻿ Thus, the objectives of this study are to develop and provide validity evidence supporting an assessment tool designed to assess the quality of educational websites in Otolaryngology- Head & Neck Surgery (ORL-HNS), and identify those that could support effective web-based learning.﻿

**Methods:**

After a literature review, the Modified Education in Otolaryngology Website (MEOW) assessment tool was designed by a panel of experts based on a previously validated website assessment tool. A search strategy using a Google-based search engine was used subsequently to identify websites. Those that were free of charge and in English were included. Websites were coded for whether their content targeted medical students or residents. Using the MEOW assessment tool, two independent raters scored the websites. Inter-rater and intra-rater reliability were evaluated, and scores were compared to recommendations from a content expert.

**Results:**

The MEOW assessment tool included a total of 20 items divided in 8 categories related to authorship, frequency of revision, content accuracy, interactivity, visual presentation, navigability, speed and recommended hyperlinks. A total of 43 out of 334 websites identified by the search met inclusion criteria. The scores generated by our tool appeared to differentiate higher quality websites from lower quality ones: websites that the expert “would recommend” scored 38.4 (out of 56; CI [34.4–42.4]) and “would not recommend” 27.0 (CI [23.2–30.9]). Inter-rater and intra-rater intraclass correlation coefficient were greater than 0.7.

**Conclusions:**

Using the MEOW assessment tool, high quality ORL-HNS educational websites were identified.

## Background

Over the past decade, there has been a proliferation of sources of medical information available in both formal and informal contexts [1, 2]. Formal platforms include scientific journals and peer-reviewed evidence-based resources (e.g., UpToDate), whereas less formal platforms may include medical education websites and lectures or tutorials available on video-sharing websites (e.g., YouTube). As evidence-based medicine (EBM) increasingly guides decision-making, access to online resources allows trainees to access up-to-date information in a timely manner [3]. Since the introduction of EBM, medical schools have gradually adopted these concepts and taught the principles of EBM to students through various methods including online instructions [3]. At the core of EBM are skills such as recognizing a knowledge gap, searching for literature and appraising the evidence [3]. Searching for pertinent and reliable medical information may thus be of particular difficulty and importance for medical students and residents, who are simultaneously acquiring medical knowledge and learning appraisal skills.

Despite such potential challenges, learners across the medical education continuum are likely to seek and appraise online resources in order to fit their learning needs and complement their formal curricula. This may particularly be true for specialties such as Otolaryngology-Head and Neck Surgery (ORL-HNS), where learning objectives and content vary significantly among medical schools [4]. Current literature within medical education research has shown that students appreciate online learning for its accessibility, ease of use, freedom of navigation, and high image quality [4, 5]. Although, there are currently multiple definitions for the term “online learning,” most authors agree that this term refers to the access of learning experiences via the use of some technology [6]. At the moment, various medical specialties have assessed web-based resources pertaining to their field, with the majority focusing on educational websites for patient teaching. However, few specialties have described educational websites available to complement formal undergraduate medical education or residency training, and a paucity of data exists in the realm of ORL-HNS.

In light of the challenges that medical trainees face when searching for reliable information and the need for complementary resources in ORL-HNS, the objectives of this study are

1. To assess the quality of educational websites in Otolaryngology- Head & Neck Surgery (ORL-HNS) using an assessment tool and identify those that could support effective web-based learning for medical students and residents.
2. To develop and provide validity evidence supporting the Modified Education in Otolaryngology Website (MEOW) assessment tool designed to assess the quality of ORL-HNS education websites.

## Methods

In order to identify high quality educational websites in Otolaryngology- Head & Neck Surgery (ORL-HNS), we engaged in a multi-stage development process:

1. Identifying and modifying a medical education website assessment tool
2. Conducting the web search to identify available websites
3. Assessing the quality of identified websites using the assessment tool
4. Providing evidence supporting validity and reliability of the assessment tool

### Identifying and modifying a medical education website assessment tool

The first step of this study was to identify a website assessment tool that could objectively identify high-quality educational websites in ORL-HNS. In order to do so, we engaged in a literature search describing medical website assessment tools using PubMed and Google Scholar. Search terms included: “medical websites evaluation tool” and “medical websites quality.” Articles highlighting the important elements of a medical education website or describing existing quality assessment tools were reviewed [2, 5–15]. Previously validated assessment tools designed to assess the quality of consumer health information websites such as the DISCERN instrument, the LIDA instrument and Health on the Net Foundation’s Health Website Evaluation tool could not be used given their limitations when applied to educational websites designed for medical trainees [13–15]. In the end, the Medical Education Website Quality Evaluation Tool (MEWQET) developed and validated for pathology websites by Alyusuf et al. was deemed to be the tool most aligned with the goal of this study. The MEWQET is in fact designed to assign a score out of 100 points by assessing 43 scoring items within 12 different categories such as authorship, content accuracy and navigability.

After identifying the MEWQET, experts in otolaryngology, medical informatics and medical education were invited to critically appraise the tool and determine its applicability to our study. After reviewing the scoring grid, it was deemed via consensus that the MEWQET could be modified to condense the website assessment process and make it more applicable to ORL-HNS websites, as several items limit its use to non-pathology related websites.

In order to modify the tool, the panel of experts reviewed each of the scoring grid’s items. Items that the authors felt were not applicable to ORL-HNS websites were discarded. Similarly, additional items important in the evaluation of medical education websites as demonstrated by the literature review were also added, including summary statements. The panel of experts also reviewed all 12 categories of the original tool. The categories were either renamed, merged or discarded. Items of the modified tool were then re-organized into the new categories.

In regards to the scoring of individual items, scores were either preserved or adapted in order to reflect their relative importance as per the original tool with input from our expert panel. Indeed, items with binary answers were attributed a maximum of 1, 2 or 3 points, and items with three possible answers were given a maximum of 2, 3 or 6 points. In the end, the total maximal score for each category also reflected the relative importance of the category as per the expert panel and the original tool.

The finalized modified version of the tool was called the Modified Education in Otolaryngology Website (MEOW) assessment tool.

### Conducting the web search to identify available websites

ORL-HNS education websites were identified using the Startpage (www.startpage.com), a Google-based search engine, to allow for reproducible search results between raters (contrary to other search engines which generate search results based on navigational history and user location). Search results were generated using the following search strategy: (“Otolaryngology” OR “head and neck surgery” OR “ENT”) AND (“resources” OR “learn” OR “educational”). The first 50 hits and all hyperlinks within these websites were analyzed. This number was decided in order to obtain the targeted pool of approximately 250 websites to be evaluated, aligned with approaches described in previous work [7]. Website inclusion criteria consisted of websites that were free of charge, in English language, targeted for ORL-HNS education, and targeted to undergraduate medical education (UGME) students/postgraduate medical education (PGME) in ORL-HNS. Websites consisting of online manuals and textbooks, journal articles, databases or search engines were excluded.

### Assessing the quality of identified websites using the assessment tool

Two raters (SH, NY) conducted the initial search to determine which websites to include and appraise in the study. The raters determined via consensus whether the website targeted: a) medical students, b) residents or c) both categories of students. This was done by reviewing the educational objectives set by the Medical Council of Canada, Royal College of Physicians and Surgeons of Canada, American Academy Otolaryngology-Head and Neck Surgery, and Accreditation Council for Graduate Medical Education (ACGME) prior to identifying content area relevant to each learner level [16–19]. Lastly, both raters used the MEOW assessment tool to assess all included websites independently twice, one week apart. Each website was thus scored four times - once by each rater on the first day, and a second time by each rater one week later. Scores were averaged across both raters on both days for all included websites in order to identify the top 3 websites for UGME, PGME and both categories.

### Providing evidence supporting construct validity and reliability of the tool

In order to provide evidence supporting construct validity of the MEOW assessment tool, scores were compared to the ratings of a content expert. This expert was Mariana Smith (MS), an outside practicing academic otolaryngologist who completed additional training at McGill University and with previous research experience. She was selected to minimize biases, as she was not involved in the development of any of the websites. Furthermore, she was blinded to the scores generated by the MEOW assessment tool. In order to provide evidence supporting validity, the rating otolaryngologist was first asked to classify 30% of websites included in this study into 1 of 3 categories: 1) Definitely recommend, 2) Maybe recommend and 3) Not recommend. These websites were randomly selected, and classification was made as per the expert’s view of the website’s educational value for medical students, residents or both. Mean MEOW assessment tool scores of websites found in each category (definitely recommend, maybe recommend, and not recommend) were compared. An analysis of variance (ANOVA) and ninety-five percent confidence intervals were calculated in order to determine statistically significant differences between the mean score of websites falling in each recommendation categories.

Website scores obtained from the MEOW assessment tool were analyzed for intra- and inter-rater reliability. Given that all websites included in this study were assessed by each rater one week apart, intra-rater reliability was measured by the scores given by the same rater to the same website. As for inter-rater reliability, mean scores obtained for each website from each rater were compared. The intraclass correlation coefficient was calculated for both intra- and inter-rater reliabilities using SPSS 20.0. The authors determined that a coefficient of more than 0.7 was the cut-off for good reliability [7].

## Results

### Tool modification

After eliminating and modifying more than 23 out of the 43 items from the previously existing assessment tool in the field of pathology, a total of 20 items blueprinted to 8 categories were included in our final assessment tool: the Medical Education in Otolaryngology Website (MEOW) assessment tool [7]. These categories included items targeted at assessing: authorship, credibility and disclosure (6 items), frequency of revision (1 item), content quality (4 items), interactivity (1 item), graphic elements (2 items), layout and design (2 items), navigability (2 items) and available hyperlinks to other resources (2 items).

In the end, the total maximal score for each category of items was considered to ensure that the proportion of points attributed to each category reflected the importance of the category as per the Medical Education Website Quality Evaluation Tool (MEWQET) with input from our expert panel. A maximum score of 56 could be attributed to a given website. The finalized version of the modified tool, the MEOW assessment tool, can be found in Table 1.

**Table 1:** The Modified Education in Otolaryngology Website (MEOW) assessment tool

Category	Criteria	Weight	Score
1. Authorship, Credibility & Disclosure	1.1 Disclosure of authorship? If yes (pick one)	No = 0	
	A. Authors’ name(s), credentials and contact information	A = 3	
	B. Authors’ name(s) with credentials	B = 2	
	C. Authors’ name(s)	C = 1	
	1.2 If author’s credentials are given, author is (if multiple authors, the majority are)		
	A. Otolaryngologist	A = 2	
	B. Other healthcare professional/scientist	B = 1	
	C. Other	C = 0	
	1.3 Disclosure of institution? If yes (pick one)		
	A. Educational, non-profit or government domain	A = 3	
	B. Other	B = 0	
	1.4 Is there an editorial review process?	Yes = 3 No = 0	
	1.5 Is the email of the webmaster provided for feedback?	Yes = 2 No = 0	
	1.6 Are references provided?	Yes = 2 No = 0	
2. Frequency of Revision	2.1 When was the website (including references) last updated?		
	A. <1 year	A = 2	
	B. ≥1 year but <5 years	B = 1	
	C. Other	C = 0	
3. Content Quality	3.1 Breadth. Does the information provided cover aspects pertinent to the field of interest?		
	A = Adequate	A = 6	
	B = Somewhat adequate	B = 3	
	C = Inadequate	C = 0	
	3.2 Depth. Is the information provided adequately detailed for the intended audience?		
	A = Adequate	A = 6	
	B = Somewhat adequate	B = 3	
	C = Inadequate	C = 0	
	3.3 Accuracy. Is the information accurate?		
	A = Accurate	A = 6	
	B = Somewhat accurate	B = 3	
	C = Inaccurate	C = 0	
	3.4 Does the website have summary statements/take-home points?	Yes = 2 No = 0	
4. Interactivity	4.1 Are there any interfaces requiring relevant action on the part of the learner (e.g., quizzes, self assessments, interactive figures)?		
	A. Definitely	A = 6	
	B. Somewhat	B = 3	
	C. No/Does not apply	C = 0	
5. Graphic Elements & Media	5.1 Are graphic/media elements included to provide additional information or to clarify existing content?		
	A. Present and pertinent	A = 2	
	B. Present	B = 1	
	C. Other	C = 0	
	5.2 Are graphic/media elements well integrated in the website?	Yes = 1 No = 0	
6. Layout & Design	6.1 Clear/professional display of available information?	Yes = 1 No = 0	
	6.2 Is the website user-friendly, having a logical layout and intuitive?	Yes = 2 No = 0	
7. Navigability & Speed	7.1 Does the website contain a search engine or table content?	Yes = 2 No = 0	
	7.2 Was the website or server accessible in a timely manner?	Yes = 2 No = 0	
8. Hyperlinks	8.1 Are there any links to provide relevant additional information?	Yes = 2 No = 0	
	8.2 If links are provided, are they active (≥90% of total links)?	Yes = 1 No = 0	

### Website assessment

**Fig. 1:**
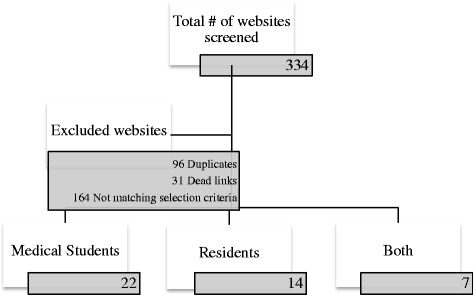
Screened, excluded and included Websites

**Fig. 2:**
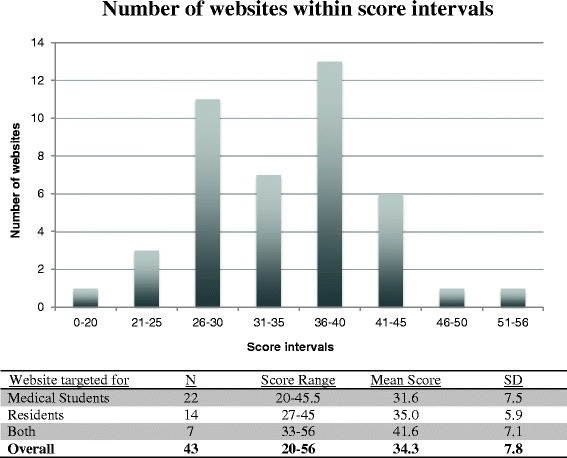
Number of websites within scores interval with summary table

**Fig. 3:**
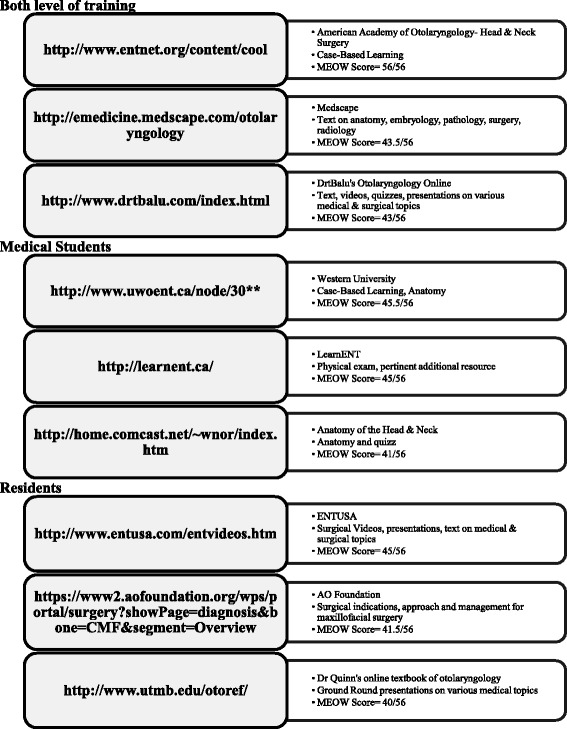
The three top scoring websites for Medical students, Residents and both level of training as evaluated using the MEOW assessment tool. **Alternative website address: http://www.schulich.uwo.ca/otolaryngology/undergraduate/clerkship.html

A total of 334 websites were identified using the search strategy described above. Of this total, 87% (291/334) were excluded (164 websites not meeting inclusion criteria, 96 website duplicates and 31 inactive links; see Fig. 1). Of the 43 included websites, 22 were considered to be targeting medical student-level educational objectives, 14 targeting resident-level objectives and seven targeting both groups. Using the MEOW assessment tool, the total scores of websites ranged from 20 to 56. The total mean score for all websites was 34.3 ± 7.8. For individual categories of websites, the mean score for websites targeted to medical students, residents and both types of learners were 31.6 ± 7.5, 35.0 ± 5.9 and 41.6 ± 7.1, respectively. The distribution of scores of all websites is demonstrated in Fig. 2. A list of the 3 websites that obtained the highest scores using the modified assessment tool for each type of learner is included in Fig. 3. The highest scoring sites included medical education websites developed by the American Academy of Otolaryngology- Head & Neck Surgery (ORL-HNS), the Canadian Society of ORL-HNS, and two Canadian universities.

### Construct validity and reliability assessments

For intra-rater reliability, intra-class correlation coefficients were 0.98 (CI [0.94–0.99]) and 0.94 (CI [0.84–0.98]) for the two raters. Regarding inter-rater reliability, intra-class correlation coefficient was 0.86 (CI [0.76–0.92]). Scores generated by the assessment tool related to the perceptions of quality made by the blinded academic otolaryngologist, with an average evaluation score of 38.4/56 (CI [34.4–42.4]) for “definitely recommend” websites, 36.2/56 (CI [33.2–39.1]) for “maybe recommend” websites and 27.0/56 (CI [23.2–30.9]) for “not recommended” websites. ANOVA analysis revealed that the differences between the means were statistically significant (*F(2, 13) = 4.13, p < 0.05)*.

## Discussion

The goal of this study was to assess and identify high quality online resources in Otolaryngology- Head and Neck Surgery (ORL-HNS) for both medical students and residents. The intent behind providing such a list of websites was to facilitate access to high quality educational content for medical trainees, and provide academic physicians with recommendable resources for their undergraduate and post-graduate programs. Indeed, given that most medical schools only offer brief clinical exposure to the specialty, students may not be inclined to purchase new reference material. Providing access to free, high-quality, readily available online material may thus enhance the learning experience. To our knowledge, no other study has previously identified such resources in the field of ORL-HNS.

Prior to this study, only one assessment tool that allows for grading of medical education websites for health professionals through a scoring system existed [7]. A standardized tool for the assessment of medical education websites arose from the need to assess and compare the quality of a website objectively and systematically rather than intuitively. As medical education websites allows for more independent learning, recognizing reliable sources of information becomes crucial for any medical practitioner. The ability to recognize and utilize high quality learning materials is a key component in evidence-based medicine and continuing professional development. Although using the Medical Education Website Quality Evaluation Tool (MEWQET) or Modified Education in Otolaryngology Website (MEOW) assessment tool may not be necessary to accomplish such appraisal, both assessment tools highlight the important components of high quality educational websites in all specialties. In fact, these tools could be of potential value to medical educators who seek to design educational websites for various medical specialties.

Our findings suggest that there may be a paucity of online resources specific to ORL-HNS with content that specifically aligns with the learning objectives set forth by national licensure committees for medical students and residents. Indeed, of the 334 websites screened, only 43 met inclusion criteria. We believe that multiple factors are at the basis of the paucity of available material. First, the use of a single search strategy rather than multiple ones, and limiting our search to the first 50 websites and their hyperlinks may have affected the total number of found websites. For instance, the University of Iowa’s head and neck website (https://iowaheadneckprotocols.oto.uiowa.edu) is a potentially useful online resource that outlines operative steps in various otolaryngology procedures that was not uncovered by the search strategy. This website was therefore not formally assessed with the MEOW assessment tool (Fig. 3). Other factors limiting the number of available websites may include the need for multiple experts (content, medical education, web development experts) and cost for educational websites development, and the perceived and actual need for educational ORL-HNS websites. Future studies should aim at determining how these websites are being used, whether they are meeting the needs of the users, and whether the number of educational websites in ORL-HNS is comparable to other medical specialties. Such information would help determine whether more educational online resources should be developed for this specialty.

When looking at the quality of available websites, scores generated by the MEOW assessment tool varied widely suggesting a spectrum in website quality. Although higher scoring websites tended to come from known organizations or institutions, one website designed by an independent otolaryngologist with unknown affiliation was highly rated. In fact, although this website did not score well in the authorship, disclosure and credibility category, the content and remainder elements resulted in an overall high score. This suggests that the modified assessment tool speaks to multiple components of quality, and how multiple factors play into the design of good education material. Nevertheless, given that raters could not be blinded to the source of websites in our study, we cannot exclude that websites from well-known organizations were unintentionally scored more favorably. This should perhaps lead us to reconsider the weight of certain authorship elements in the conceptualization of ‘credible’ resources for online learning.

Our study has several limitations. In addition to the limitations pertaining to our search strategy and potential selection bias due to raters’ knowledge of the websites’ source, inter-rater reliability may have been affected given that both raters worked collaboratively to include or exclude screened websites. Scores generated by the MEOW tool were significantly different between websites that were rated as “recommend” and “maybe recommend” by an expert ORL-HNS educator from those that were rated as “not recommend.” However, although there seems to be a trend, no statistically significant difference was demonstrated between websites’ scores in the first two categories. While, this may be due to the relative small sample size, such finding may suggest that the MEOW assessment tool is most useful in differentiating high and average quality websites from lower quality ones.

## Conclusions

Online learning resources constitute integral sources of information for today’s learners, but are associated with variable quality. The Modified Education in Otolaryngology Website (MEOW) assessment tool has been shown to be a validated instrument that can objectively assess educational websites for medical trainees. With its application, we were able to identify high-quality educational websites pertaining to the field of ORL-HNS that could enhance the learning experience of medical trainees.

## Abbreviations

ACGME: Accreditation Council for Graduate Medical Education
EBM: Evidence-based medicine
MEOW: Modified Education in Otolaryngology Website
MEWQET: Medical Education Website Quality Evaluation Tool
ORL-HNS: Otolaryngology- Head & Neck Surgery
PGME: Postgraduate medical education
UGME: Undergraduate medical education
